# Can Zika Account for the Missing Babies?

**DOI:** 10.3389/fpubh.2017.00317

**Published:** 2017-11-29

**Authors:** Flávio Codeço Coelho, Margaret Armstrong, Valeria Saraceni, Cristina Lemos

**Affiliations:** ^1^Center for Mathematical Epidemiology, School of Applied Mathematics, Fundação Getúlio Vargas, Brasília, Brazil; ^2^Prefeitura da Cidade do Rio de Janeiro, Rio de Janeiro, Brazil

**Keywords:** Zika, sexually transmitted diseases, infant, newborn, diseases, live birth rate, epidemiology

## Abstract

The Zika virus (ZIKV) spread rapidly in Brazil in 2015 and 2016. Rio de Janeiro was among the Brazilian cities which were hit the hardest, with more that a hundred thousand confirmed cases up to the end of 2016. Given the severity of the neurological damage caused by ZIKV on fetuses, we wondered whether it would also cause an increase in the number of miscarriages, especially very early ones. As early miscarriages are unlikely to be recorded as a health event, this effect—if it occurred—would only show up as a reduction in the number of live births. In this article, we show that there was a 15% drop in live births between September and December 2016 compared with the previous year, and that this sharp drop from epidemiological week 33 onward is strongly correlated with the number of recorded cases of Zika about 40 weeks earlier. We postulate that ZIKV is directly responsible for this drop in the birth rate. Further work is required to ascertain whether other factors such as the fear of having a microcephaly baby or the economic crisis are having a significant effect.

## 1 Introduction

Propelled by a combination of vectorial and sexual transmission, the Zika virus (ZIKV) spread rapidly in Brazil in 2015 and 2016, with the epidemic reaching neighboring countries very quickly (1). Rio de Janeiro was among the Brazilian cities which were hit the hardest. With more than 6 million inhabitants, it is an excellent place to observe effects which would be hard to detect in smaller populations. It was based on data from Rio that the increased risk of Zika in females of reproductive age was first described (2).

The data on the Zika epidemic in Rio still keep revealing important new facts about this emerging infection. In a recent publication, de Oliveira et al. (3) commented that the data collected cannot explain why there are much fewer cases of microcephaly than expected in 2016, whereas the number of cases Guillain–Barré syndrome (GBS) was more or less as expected. They propose three possible reasons for this: (1) the increase in the cases of GBS was due to another virus such as CHIKV rather than ZIKV and cases of fever were wrongly attributed to ZIKV; (2) ZIKV is a necessary but not sufficient cause for microcephaly; and (3) fear of adverse consequences led to fewer conceptions.

In this article, we propose a fourth possibility: ZIKV causes miscarriages early in pregnancy—even before the mother realizes that she is pregnant (4, 5). To demonstrate this, we combine evidence from Zika incidence and the birth records. As early miscarriages are unlikely to be recorded as a health event, this effect—if it occurred—would only show up as a reduction in the number of live births. We conclude that one can indeed see a statistically significant drop in the number of live births which can be traced back to Zika incidence roughly 40 weeks earlier.

## 2 Materials and Methods

### 2.1 Data

Data about live births from the city of Rio de Janeiro were obtained from SINASC, the national system for live births registration. The birth rate in Rio has been stable for a number of years.

The reported cases of Zika were obtained from SINAN, the national registry of diseases of mandatory reporting. Reporting of Zika became mandatory in late 2015. We only had access to SINAN and SINASC data from the city of Rio de Janeiro.

### 2.2 Model

As births show a natural annual seasonality, we set out to measure the loss in births on a week-by-week basis, in response to the incidence of Zika. To further strengthen the hypothesis of causal association between the Zika incidence and the drop in births, we also included the incidence of Chikungunya in the model as well.

Let $B_{t}^{2015}$ be the number of birth live births on week *t* of 2015. Let $D_{t}=B_{t}^{2015}-B_{t}^{2016}$ for *t* ranging from 1 to 52, the number of weeks in 1 year. Let *Z_t_* and *C_t_* be the number of female Zika and Chikungunya cases notified on week *t*, respectively. Assuming *D_t_* follows a Gaussian distribution, we proposed the following generalized linear model:

(1) Dt=β0+β1log(Zt−τ)+β2log(Ct−τ)+ϵ,

(2) ϵ∼N(0,σ2),

where *τ* is in weeks and takes values from the interval (33, 45). This model tests the hypothesis that the incidence of Zika *τ* weeks earlier is positively correlated with the deficit of births in week *t*, defined above and denoted by *D_t_*.

## 3 Results

**Figure 1:**
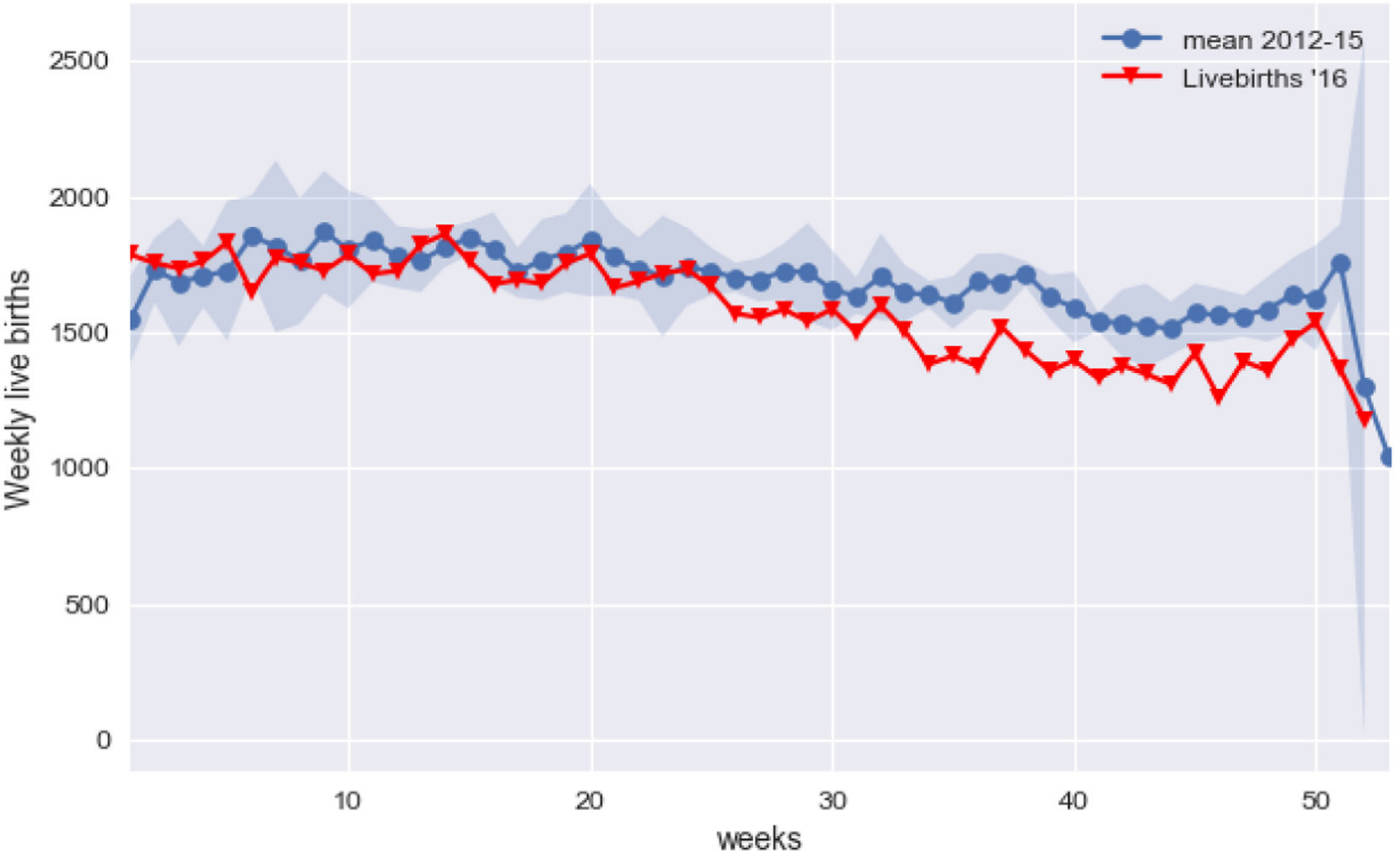
Average births per week for year 2012–2015 (in blue) and weekly births in 2016.

Figure 1 shows the number of live births per week in 2016 in red compared with the average number per week from 2012 to 2015 in blue with the 95% confidence shaded around it. From week 33 onward (i.e., about 40 weeks after the start of the ZIKV epidemic), the red line is consistently below the confidence interval.

**Figure 2:**
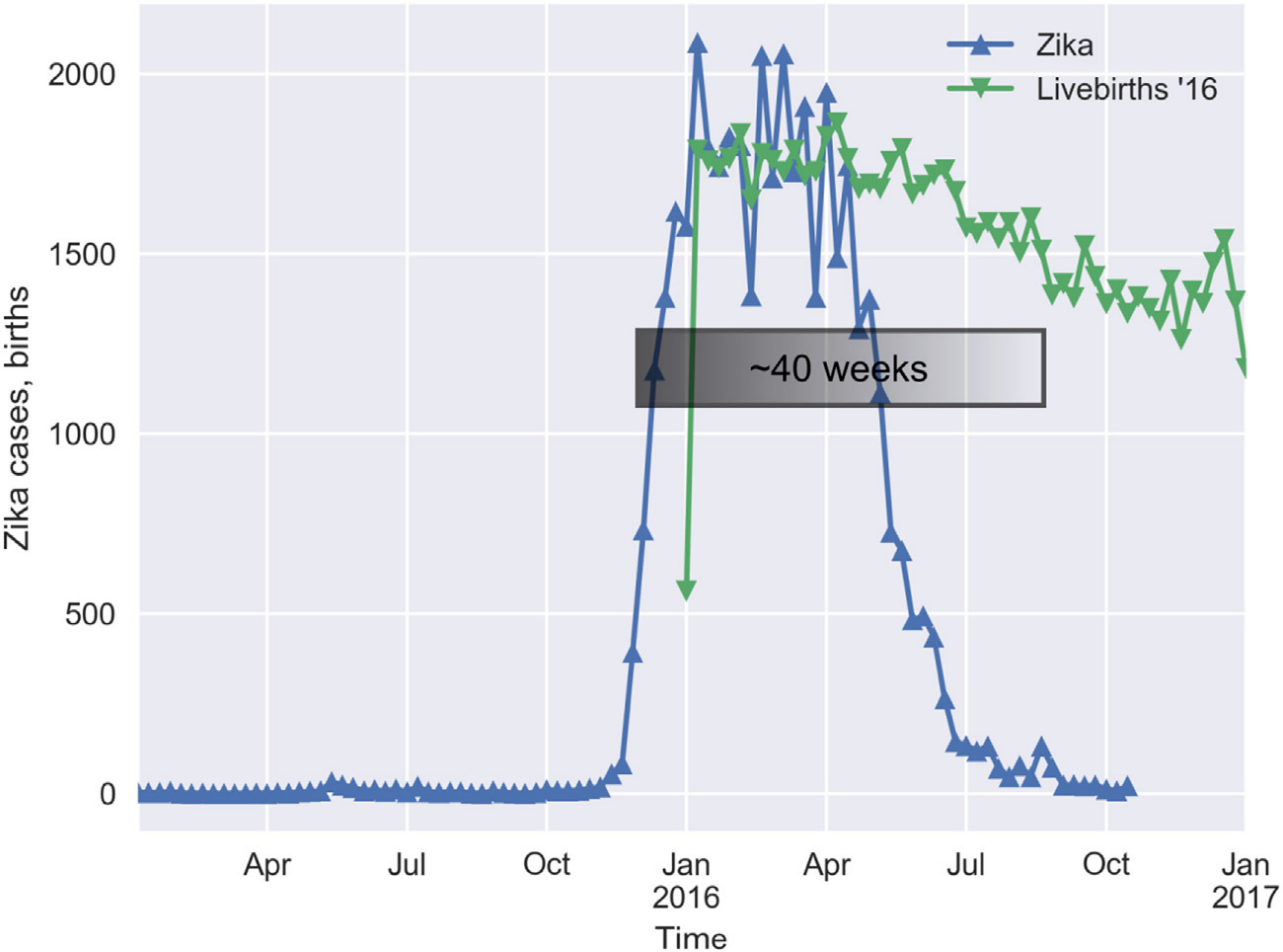
The lag between the onset of the Zika outbreak and the increase in the loss of births is roughly 40 weeks.

Taking the average number of births from 2012 to 2015 as the expected number for 2016, there were 5,154 missing babies, from week 33 on. This figure represents a 14.85% drop in expected number of births for this period. The number of missing babies for the entire year was 7,484, so the bulk of the deficit in births really occurred after week 33 (Figure 2).

The results in Table 1 show that the weekly loss of births is statistically associated with the incidence of Zika in the past. The association is stronger in the lag-time window of 38–42 weeks (*p* < 0.01). Weaker but still significant (*p* < 0.05) association can be seen in the range of 35–37 and at 43 weeks after the Zika epidemic.

**Table 1:** Results of fitting the model to the data for *τ* ranging from 33 to 45.

*τ* (weeks)	*β*_1_	*β*_2_	p-Values: *log*(*Z*_t−_*_τ_*), *log*(*C*_t−_*_τ_*)
33	15.56	6.83	0.11, 0.82
34	10.28	30.29	0.27, 0.30
35	21.93	−16.45	0.02, 0.57
36	20.78	−2.88	0.02, 0.91
37	17.36	8.54	0.05, 0.76
**38**	**24.23**	−15.7	**0.007**, 0.57
**39**	**24.24**	−5.55	**0.005**, 0.83
**40**	**25.27**	−10.29	**0.001**, 0.67
41	18.58	7.13	0.022, 0.77
**42**	**29.24**	−34.96	**0.000**, 0.162
43	17.99	7.74	0.03, 0.76
44	15.38	13.16	0.07, 0.62
45	23.94	−47.85	0.025, 0.14

*Figures in bold are significant at 1%*.

## 4 Discussion

Although the analysis presented does not completely rule out other influences on Rio de Janeiro’s birth rate, it demonstrates a strong correlation between the drop in the birth rate and the number of Zika cases. The actual range of lags of the influence and the lack of an association with Chikungunya, which happened at approximately the same period, points strongly to Zika being a causal factor in this loss of babies.

Zika virus has demonstrated the ability to cross the placental barrier and cause congenital disease both in animal models and in humans (6, 7). The implications of a ZIKV infection during pregnancy for the health of fetuses are quite well established (8), but the impact of Zika on early miscarriages is still anecdotal (4). Johansson et al. (9) argue that the risk of microcephaly greater if the ZIKV infection happens during the first trimester of pregnancy. Combining their evidence with ours, we conclude that microcephaly cases would be just the tip of the iceberg, since most of the first-trimester infections would lead to miscarriages instead. Further studies might be able to use microcephaly incidence rates to help estimate unreported fetal loss.

We hope the evidence provided by this study will lead to more specific investigations to determine if miscarriages after a Zika epidemic can be traced back to infections during pregnancy. The drop in the birth rate, even if only due to family planning, would still be a serious consequence of a Zika epidemic, because of its economic implications.

## Author Contributions

FC and MA conceived the work and analyzed the data. VS and CL provided the data and helped to develop the discussion of the results. All the authors contributed to writing of the manuscript.

## Conflict of Interest Statement

The authors declare that the research was conducted in the absence of any commercial or financial relationships that could be construed as a potential conflict of interest.

## References

[1] Fauci AS Morens DM Zika virus in the Americas – yet another arbovirus threat. N Engl J Med (2016) 374(7):601–4. 10.1056/NEJMp1600297 26761185

[2] Coelho FC Durovni B Saraceni V Lemos C Codeço CT Camargo S Sexual transmission causes a marked increase in the incidence of Zika in women in Rio de Janeiro, Brazil. bioRxiv (2016) 055459 10.1101/055459 27664930

[3] de Oliveira WK Carmo EH Henriques CM Coelho G Vazquez E Cortez-Escalante J Zika virus infection and associated neurologic disorders in Brazil. N Engl J Med (2017) 376(16):1591–3. 10.1056/NEJMc1608612 28402236 PMC5544116

[4] van der Eijk AA van Genderen PJ Verdijk RM Reusken CB Mögling R van Kampen JJ Miscarriage associated with Zika virus infection. N Engl J Med (2016) 375(10):1002–4. 10.1056/NEJMc1605898 27463941

[5] Brasil P Pereira JP Raja Gabaglia C Damasceno L Wakimoto M Ribeiro Nogueira RM Zika virus infection in pregnant women in Rio de Janeiro – preliminary report. N Engl J Med (2016) 375:2321–34. 10.1056/NEJMoa1602412 26943629 PMC5323261

[6] Vermillion MS Lei J Shabi Y Baxter VK Crilly NP McLane M Intrauterine zika virus infection of pregnant immunocompetent mice models transplacental transmission and adverse perinatal outcomes. Nat Commun (2017) 8:14575. 10.1038/ncomms14575 28220786 PMC5321801

[7] Cao B Diamond MS Mysorekar IU. Maternal-fetal transmission of Zika virus: routes and signals for infection. J Interferon Cytokine Res (2017) 37(7):287–94. 10.1089/jir.2017.0011 28402153 PMC5512303

[8] Teixeira MG da Conceição N Costa M de Oliveira WK Nunes ML Rodrigues LC. The epidemic of Zika virus-related microcephaly in Brazil: detection, control, etiology, and future scenarios. Am J Public Health (2016) 106(4):601–5. 10.2105/AJPH.2016.303113 26959259 PMC4816003

[9] Johansson MA Mier-y Teran-Romero L Reefhuis J Gilboa SM Hills SL Zika and the risk of microcephaly. N Engl J Med (2016) 375(1):1–4. 10.1056/NEJMp1605367 27222919 PMC4945401

